# Extracting Group Velocity Dispersion values using quantum-mimic Optical Coherence Tomography and Machine Learning

**DOI:** 10.1038/s41598-023-32592-7

**Published:** 2023-04-22

**Authors:** Krzysztof A. Maliszewski, Magdalena A. Urbańska, Piotr Kolenderski, Varvara Vetrova, Sylwia M. Kolenderska

**Affiliations:** 1 School of Mathematics and Statistics, University of Canterbury, Christchurch, New Zealand; 2 Massey AgriFood (MAF) Digital Lab, Massey University, Palmerston North, New Zealand; 3 Faculty of Physics, Astronomy and Informatics, Nicolaus Copernicus University, Toruń, Poland; 4 School of Physical and Chemical Sciences, University of Canterbury, Christchurch, New Zealand

**Keywords:** Mathematics and computing, Physics

## Abstract

Quantum-mimic Optical Coherence Tomography (Qm-OCT) images are cluttered with artefacts - parasitic peaks which emerge as a by-product of the algorithm used in this method. However, the shape and behaviour of an artefact are uniquely related to Group Velocity Dispersion (GVD) of the layer this artefact corresponds to and consequently, the GVD values can be inferred by carefully analysing them. Since for multi-layered objects the number of artefacts is too high to enable layer-specific analysis, we employ a solution based on Machine Learning. We train a neural network with Qm-OCT data as an input and dispersion profiles, i.e. depth distribution of GVD within an A-scan, as an output. By accounting for noise during training, we process experimental data and estimate the GVD values of BK7 and sapphire as well as provide a qualitative GVD value distribution in a grape and cucumber. Compared to other GVD-retrieving methods, our solution does not require user input, automatically provides dispersion values for all the visualised layers and is scalable. We analyse the factors affecting the accuracy of determining GVD: noise in the experimental data as well as general physical limitations of the detection of GVD-induced changes, and suggest possible solutions.

## Introduction

Quantum-mimic Optical Coherence Tomography (Qm-OCT) achieves resolution enhancement and even-order dispersion cancellation by mimicking quantum entanglement found in Quantum OCT. Proposed theoretically in various forms,^1–3^ Qm-OCT is realised experimentally by introducing modifications in the OCT detection setup^4–7^ or simply by applying a computer algorithm to raw OCT spectra^8^,^9^. In principle, a Qm-OCT A-scan is obtained by Hilbert-transforming the spectrum, autocorrelating it and then performing Fourier transformation. A signal which is much more useful in terms of the information content is called an FFT stack and is obtained by processing several fragments of the spectrum.

The core of this method is autocorrelation whose implementation - either experimental or algorithmic - results in the creation of artefacts. Artefacts are additional peaks which do not represent the structure of the imaged object and lead to the image scrambling for multi-layered objects. These artefacts are layer-specific: their behaviour and shape in the FFT stack are related to the optical parameters of the layer they correspond to. One of such optical parameters is Group Velocity Dispersion (GVD) representing wavelength-dependent variations of the refractive index inside an object. GVD is considered as detrimental since it leads to resolution degradation, especially for deeper layers, due to dispersion’s accumulative nature and inability to compensate for it for each individual layer at once. However, this detrimental effect can be used to one’s advantage: GVD values can be extracted to characterise the imaged object. In general, methods enabling GVD extraction make use of the following effects of dispersion on the signal: resolution degradation^10,11^, peak location shift between A-scans obtained from two different fragments of a spectrum^12^, and spectral phase differences^13^. A very good comparison of these methods’ performance could be found in the publication by Photiou and Pitris^14^. Extracted GVD can be correlated with the salinity of water-like media^15^ or can even be correlated with early signs or progression of diseases^11^. Unfortunately, the current approaches used to determine GVD values are either very error-prone^11^, or work only for very simple objects^10,12^. Although it was shown that in some instances they achieve average errors of 1%^14^, they are far from being automatic and require user input, especially when GVD is to be retrieved for multiple layers within an A-scan.

It was shown that Machine Learning^16^ - when combined with Qm-OCT - enables to estimate qualitatively GVD value distribution, i.e. a dispersion profile, within an A-scan. Here, we propose an improved solution that accounts for noise in the data. Our approach is tested on two experimental systems, a laboratory and commercial one, and allows a successful determination of GVD values from experimental data for sapphire and BK7 and provides qualitative dispersion profiles of a grape and cucumber. The limitations of our approach in terms of the noise levels, optical parameters mismatch and detectable GVD values are discussed.

## Data

**Figure 1:**
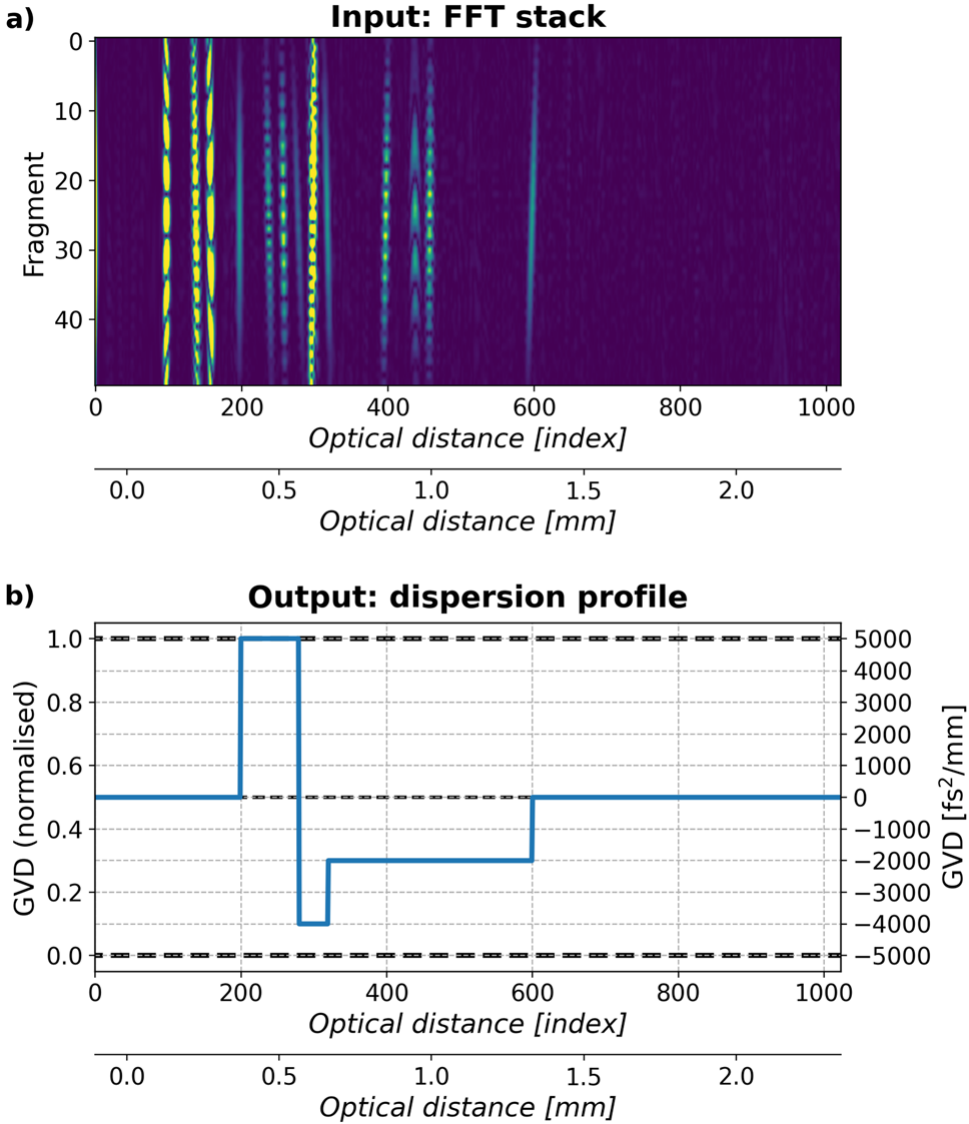
(**a**) The input of the neural network is an FFT stack which is calculated from a raw experimental spectrum. The training FFT stacks are calculated from computer-generated spectra. (**b**) The output is a dispersion profile, i.e. the GVD value distribution within the object in an A-scan. GVD equal to 0 fs$^2$/mm corresponds to 0.5 in the dispersion profile, and the maximum and minimum GVD values used for training, 5000 and −5000 fs$^2$/mm correspond to 1 and 0 in the dispersion profile. The presented object has four interfaces placed at the positions 200, 280, 320 and 600 corresponding to the distance from zero optical distance equal to 218 μm and the object layers thicknesses of 87, 44 and 306 μm. The GVD value is 0 fs$^2$/mm for the area in front of the object and 5000, $-4000$, and $-2000$ fs$^2$/mm for the object layers.

We use entirely computer-generated data: noisy Qm-OCT signals as inputs and dispersion profiles as outputs. An input Qm-OCT signal (Fig. 1a) is created with the algorithm in Ref.^9^: first a 1024 element-long spectrum is synthesised (at central wavelength of 840 nm, and with the total spectral bandwidth of 160 nm, a Gaussian profile and Gaussian noise, resulting in the axial resolution equal to 4.08 μm), then split into 50 fragments, which are then autocorrelated, zero-padded to be 2048 element-long and Fourier transformed. Half of a Fourier transform is taken, which means that the resultant signal size is 50 by 1024 elements. The output dispersion profiles (Fig. 1b) are 1024-element-long vectors whose elements are in the range [0,1] corresponding to the GVD value range of (−5000, 5000) fs$^2$/mm. As presented in Fig. 1b, a dispersion profile consists of constant-value segments whose location corresponds to the location of a layer and whose height represents layer’s GVD. This does not necessarily mean that the GVD distribution within a particular layer is perfectly constant. Since GVD of a layer in OCT A-scans manifests itself by broadening the peak corresponding to the back-surface of that layer, one observes the dispersion accumulated between the front and back surface of the layer. This is why, the height of the segment corresponds only to the GVD behind the back-surface peak broadening.

We generated 260,000 object parameters for training, 20,000 for validation and 20,000 for testing. The generated object parameters are used in the on-fly calculation of the training, validation and test datasets: due to the processing limitations of the computer, the input and ground truth signals are calculated anew in every epoch.

The experimental data was obtained with a laboratory setup and a commercial system. The laboratory OCT system best matches the optical parameters simulated in the training datasets. It uses a light source (Superlum Broadlighter T840-HP) with the central wavelength of 840 nm and the total spectral bandwidth of 160 nm, and an Optical Spectrum Analyser (Yokogawa AQ6370D) as a spectrometer which outputs 2048-point long spectra with the spectral resolution of 0.1 nm. The axial resolution in air is 4.1 μm and the 6-dB fall-off 1.4 mm. The commercial system is OQ LabScope 2.0 /SX using light with the central wavelength of 830 nm and the total spectral bandwidth of 100 nm (resulting in the axial resolution in air of 5.5 μm and imaging range of 1.2mm) and detection based on a spectrometer that produces 2048-point long spectra with a rate of 40,000 A/scans per second. The grape and onion used in this work were purchased in the local supermarkets.

## Neural network

**Figure 2:**
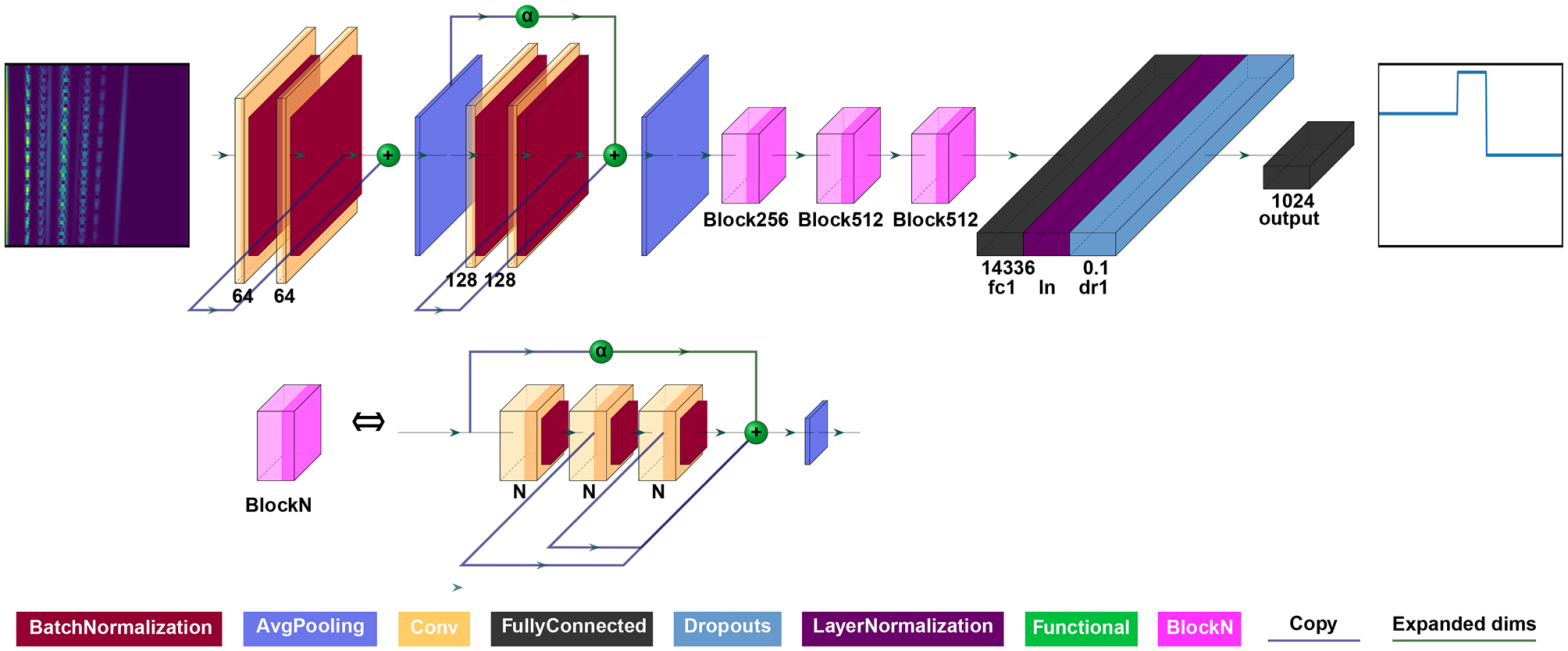
Model architecture. Blue lines are the copies of layers’ outputs. Green lines represent alpha layer (symbolised by the green dot with $\alpha$ character) output with an expanded shape to match the shape of the convolutional layers inside the block. BlockN (pink square) is a block with convolutional layers each consisting of N filters. The plot was created with the help of PlotNeuralNet software^18^.

We cast this problem as a Machine Learning problem with an FFT stack as an input and a dispersion profile as an output. Our neural network is based on a modified VGG-16 architecture^17^ which we showed is capable of interpreting layer-specific behaviour and output a depth-resolved dispersion profile of the objects^16^. In the modified architecture, batch normalization is added after each convolutional layer and residual blocks are used to sum up the outputs of the pooling layer and convolutional layers. Figure 2 shows the final architecture of the model. A more detailed description of the network parameters is found in Section 1 in Supplement 1.

Our optimised model architecture consists of batch normalization^19^ layer after each convolutional layer and each convolutional block is followed by an average pooling layers, one fully connected layer with 14336 units and dropout rate^20^ of 0.1 followed by a layer normalization^21^. We trained the models with batch size 16, learning rate 0.0001, optimizer Adam^22^, loss function Mean Absolute Error and sigmoid as output activation function.

Training of our models was performed on a computer with NVIDIA GeForce GTX 3060 12GB graphics card. We trained our models for at least 100 epochs. On-fly data generation took most of 1.5 h for each epoch to finish. The analysis of the training process and performance is presented in Sections 2–4 in Supplement 1.

## Results

### Signal to noise ratio

**Figure 3:**
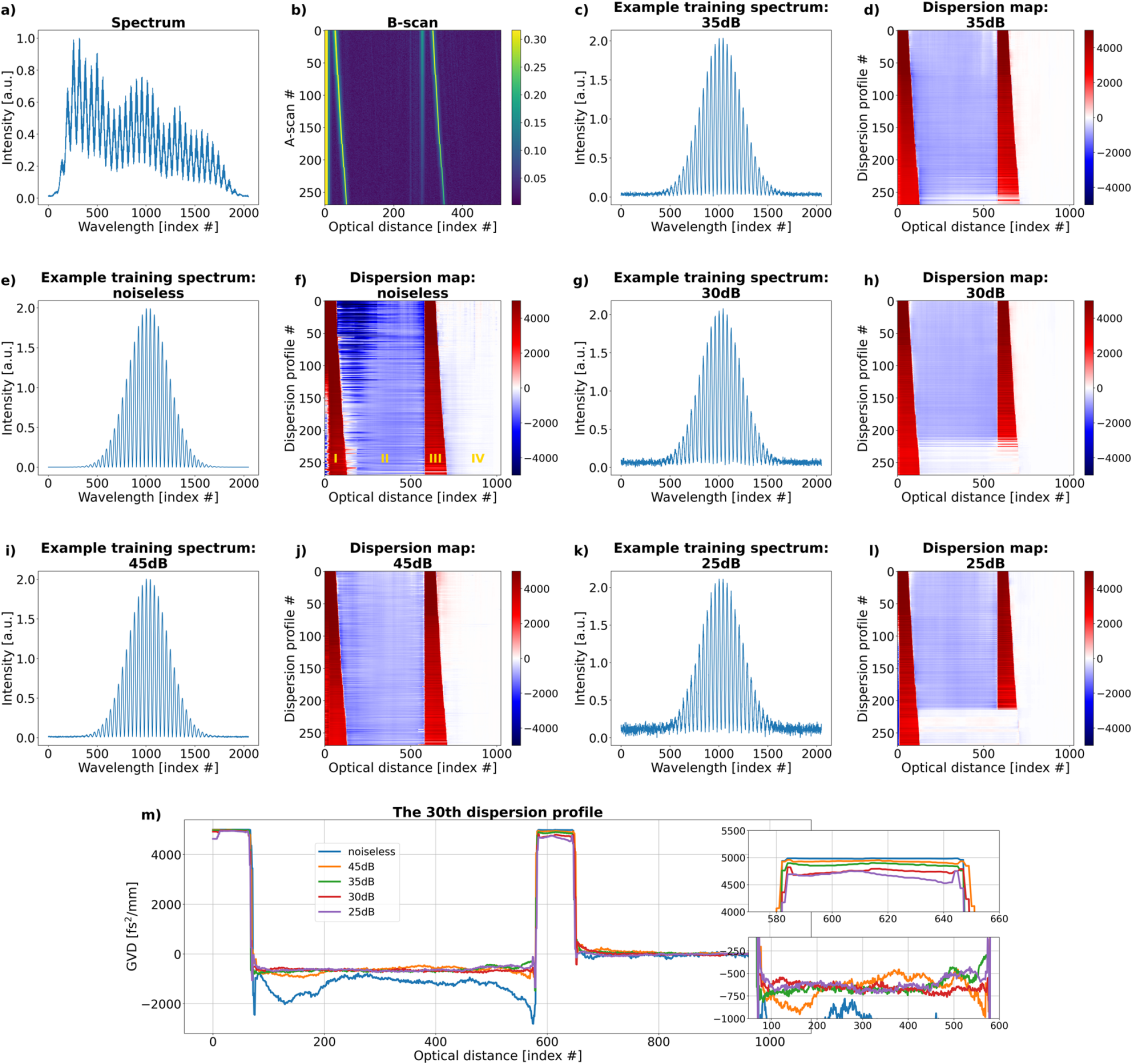
(**a**) One of the experimental spectra acquired for a 460-μm thick sapphire. (**b**) A B-scan showing a tilted sapphire glass. The vertical line represents autocorrelation peaks. (**f**, **j**, **d**, **h**, **l**) Dispersion maps of the sapphire glass obtained with a network trained with signals containing: no noise, 45, 35, 30 and 25 dB noise. All the dispersion maps are affected by the presence of the autocorrelation peaks: the network treats them as structural peaks and assigns GVD levels to them. I–IV mark four areas that can be discerned as a result. (**c**, **e**, **g**, **i**, **k**) Example training spectra showing what the simulated noise levels look like. (**m**) The 30th dispersion profile from all five predicted dispersion maps.

The neural networks were trained using datasets comprising of signals with different levels of noise: 45, 35, 30 and 25 dB and tested on experimental data in order to assess which noise level is most optimal. To give a sense of what each noise level looks like in a training signal, example spectra were generated for a single-interface object and with the maximum modulation depth. They are presented in Fig. 3e, g, i and k, with Fig. 3c showing a spectrum with no noise.

The experimental dataset, obtained using the laboratory OCT system, contains 275 FFT stacks calculated from 275 experimental spectra that were obtained while transversely scanning a 460-μm thick sapphire glass. The spectra - one of which is shown in Fig. 3a - are linearised to remove the nonlinearity introduced by the spectrometer, filtered with a Gaussian window, and then Fourier transformed. The resulting A-scans are stacked one on top of another to form an XZ image called a B-scan presented in Fig. 3b. Because the sapphire was placed at an angle to the light propagation direction, the front and back surfaces of the sapphire are represented by two tilted lines. The vertical line located near the second sapphire surface corresponds to autocorrelation peaks. The autocorrelation peaks are inherent to OCT and originate from the interference of light reflected from the internal structures of the object. Because sapphire is a two-interface object, every A-scan contains one autocorrelation peak placed at a distance equal to the optical thickness of the sapphire.

The sapphire dataset is processed with a network trained with noiseless data and networks trained with noisy data. Each FFT stack from the dataset is transformed by a network into a dispersion profile. All the dispersion profiles are stacked one on top of another to form a dispersion map. Such predicted dispersion maps are shown in Fig. 3d corresponding to “noiseless” training and Fig. 3f, h, j and l corresponding to training with the noise levels of 45, 35, 30 and 25 dB, respectively.

Since the autocorrelation peak is treated by the neural network as a structural peak (as discussed in^23^, the dispersion profiles contain four distinct areas (marked with yellow I–IV in Fig. 3f), instead of expected three:

1. Area I starts at 0 optical distance and ends at the location of the first interface. The GVD in this area should be equal to the GVD of the dispersion imbalance in the interferometer, $\beta _{NL,\text {front}}^{(even)}$.
2. Area II starts at the location of the first interface and ends at the location of the autocorrelation peak. The GVD in this area, $\beta _{NL, 2}^{(even)}$, depends on the GVD of the first area and the object GVD, $\beta _{NL,obj}^{(even)}$^23^
  1 $$\begin{aligned} \beta _{NL, 2}^{(even)} = \frac{ L_{\textrm{front}} \beta _{NL, \textrm{front}}^{(even)} - L_{obj} \beta _{NL, obj}^{(even)} }{L_{\textrm{front}} - L_{obj}}, \end{aligned}$$
  where $L_{\textrm{front}}$ is the distance between 0 optical distance and the first object interface, and $L_{obj}$ is the object thickness.
3. Area III, situated between the autocorrelation peak and the second interface, should have the same GVD as area I.
4. In the fourth area, area IV, located behind the second interface, the GVD is equal to 0 fs$^2$/mm.

All of the predicted dispersion maps contain all four areas, with areas I and III having a similar GVD value. It can be noticed that the more “noisy” the training, the better the predictions: the GVD values within all four areas become more uniform and the areas themselves smoother, with the smoothest map being the output of the 30dB network. To see that improvement on the level of a single dispersion profile, the 30th dispersion profile of the five predicted dispersion maps is plotted and shown in Fig. 3m. Also, in the predicted dispersion maps for 35dB, 30dB and 25dB networks, three areas are detected, instead of four, on the bottom of the maps: one between 0 optical distance and the first interface, one between the interfaces, and the third one behind the second interface. This is due to the fact that at these noise levels, the network treats the autocorrelation peak as noise and assigns GVD levels to just two peaks (the interface peaks), instead of three (the interface peaks and the autocorrelation peak).

The network trained on signals with signal-to-noise ratio (SNR) equal to 30dB was chosen to be used further, as it visibly shows the best results: the dispersion map is the “smoothest”. More detailed and parameter-focused analysis and comparison of the performance of the model trained with datasets corresponding to different SNR levels are presented in Section 3 in Supplement 1.

### Resolution mismatch

It was tested how the neural network predictions are affected if the axial resolution of the input FFT stacks used for training, 4.08 μm, does not match the axial resolution of the OCT system.

**Figure 4:**
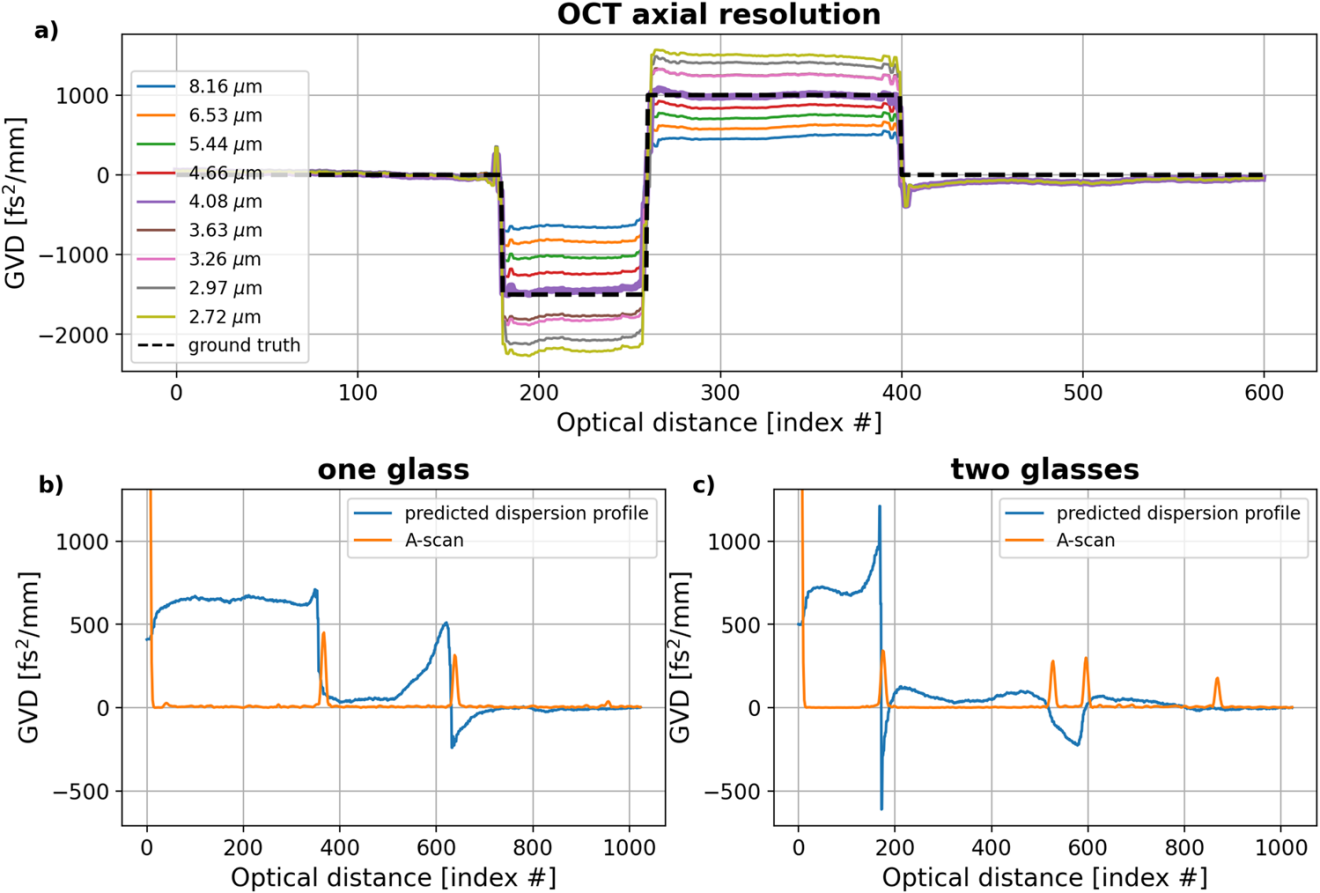
Predicted dispersion profiles for a three-interface object with GVD of -1,500 fs$^2$/mm and 1,000 fs$^2$/mm corresponding to (**a**) different OCT system resolution values show the network’s potential for universal use if the resolution mismatch is accounted for. Predicted dispersion profiles in blue, with corresponding A-scans in orange, for (**b**) one 120-μm thick quartz glass (**c**) two 120-μm thick quartz glasses show similar GVD levels inside the objects and in front of the objects.

FFT stacks for a three-interface object with GVD of −1500 fs$^2$/mm and 1000 fs$^2$/mm and corresponding to different axial resolutions, 2.72 to 8.16 μm, were generated and processed with the neural network. As shown in Fig. 4a, the predicted GVD level changes proportionally to the axial resolution: the smaller the resolution value, the smaller the predicted GVD and the bigger the resolution value, the bigger the predicted GVD.

The obtained predictions show that the network could be used with data acquired with an OCT system whose axial resolution does not match the ones used for training. In the presence of mismatch, the results are qualitative, and the influence of a mismatched resolution could potentially be corrected by applying a proportionality constant to get a more quantitative representation.

Two kinds of objects were imaged with the commercial system for which the mismatch between the experimental resolution (5.5 μm) and the simulated resolution (4.08 μm) is present: one 120-μm thick quartz glass and two 120-μm thick quartz glasses of the same kind (A-scans presented in Fig. 4b and c in orange). The predicted dispersion profiles (Fig. 4b and c in blue) show similar GVD levels for the inside of the glasses, around 25 fs$^2$/mm, and between 0 optical distance and the location of the first object interface, 680 fs$^2$/mm. The GVD levels are not constant within the corresponding areas and show rapid changes at boundaries - errors being the current limitation of our approach. However, the GVD levels heights look fairly consistent in the two dispersion profiles: the GVD level for the inside of the quartz glasses and around 0 optical distance are similar in both dispersion profiles.

### GVD levels variability within the object

**Figure 5:**
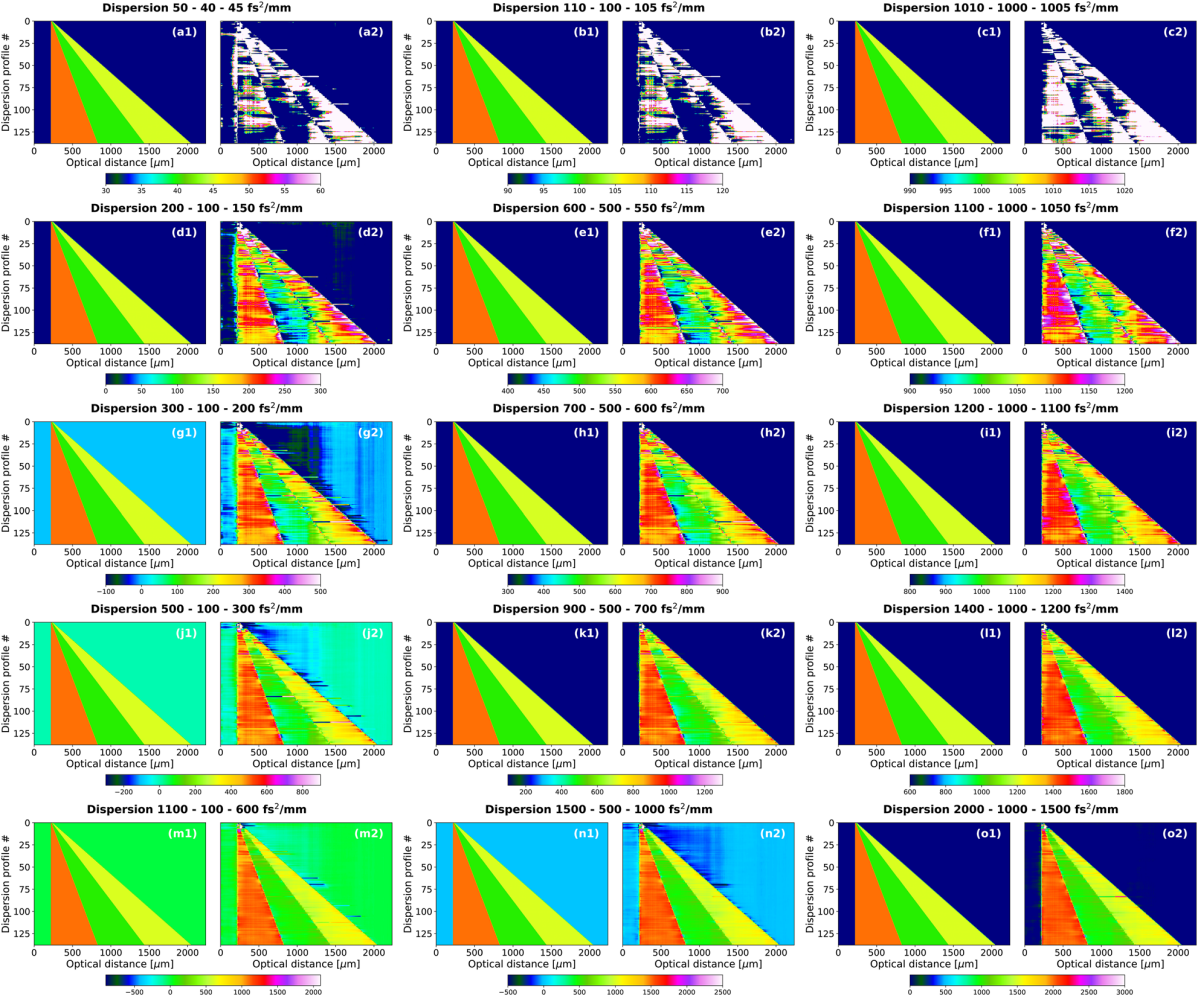
(**a**1–**o**1) Ground truth dispersion maps, (**a**2–**o**2) Dispersion maps predicted using the 30 dB neural network. The titles of each pair of images state the GVD values of the layers of the object: the values increase from left to right in each row and the difference of GVD values within the object increases from top to bottom. The greater the variability of GVD values within the object, the better the predictions.

The neural network was tested in terms of prediction limitations with computer-generated signals representing three-layered objects for which all layers are of equal thickness and where each layer has a different GVD value (Fig. 5). The graphs in Fig. 5 labeled with 1 are the ground truth dispersion maps with 138 dispersion profiles each. The consecutive dispersion profiles in a dispersion map represent an increasing thickness of the object layers: 4 μm thickness of all three layers for the 0th dispersion profile and 306 μm thickness of all three layers for the 137th dispersion profile. The graphs in Fig. 5 labeled with 2 are predictions.

The first row in Fig. 5 shows the most realistic situations in the terms of the GVD values and their layer-to-layer variability in the object. The GVD values differ by 10 and 5 fs$^2$/mm between the layers of the object, resulting in: 50, 40, 45 fs$^2$/mm in Fig. 5a, 110, 100 and 105 fs$^2$/mm in Fig. 5b, and 1010, 1000 and 1005 fs$^2$/mm in Fig. 5c. The variability of GVD levels is higher in each consecutive row: in Fig. 5d, e the difference is 100 and 50 fs$^2$/mm, in Fig. 5g–i 200 and 100 fs$^2$/mm, in Fig. 5j–l 400 and 200 fs$^2$/mm and in Fig. 5m–o 1000 and 500 fs$^2$/mm with the minimum levels of GVD in each row being 100, 500 and 1000 fs$^2$/mm (corresponding to the middle layer).

The predictions of dispersion maps of objects with the least GVD variability fail to reproduce the correct values, but still show sharp changes at the locations of the interface, and therefore enable one to distinguish the layers. The larger the GVD variability is within the object, the better the predictions are, even for thicknesses as small as couple of micrometers. This behaviour is not surprising. Smaller layers and/or smaller GVD do not induce a change of the artefacts in the FFT stack big enough for the neural network to pick up, on the other hand, thicker layers and/or bigger GVD introduces a greater change in the appearance of the FFT stack making it much easier for the neural network to interpret.

A more quantitative and parameter-oriented analysis is found in Section 4 in Supplement 1.

### GVD value predictions for sapphire and BK7

**Figure 6:**
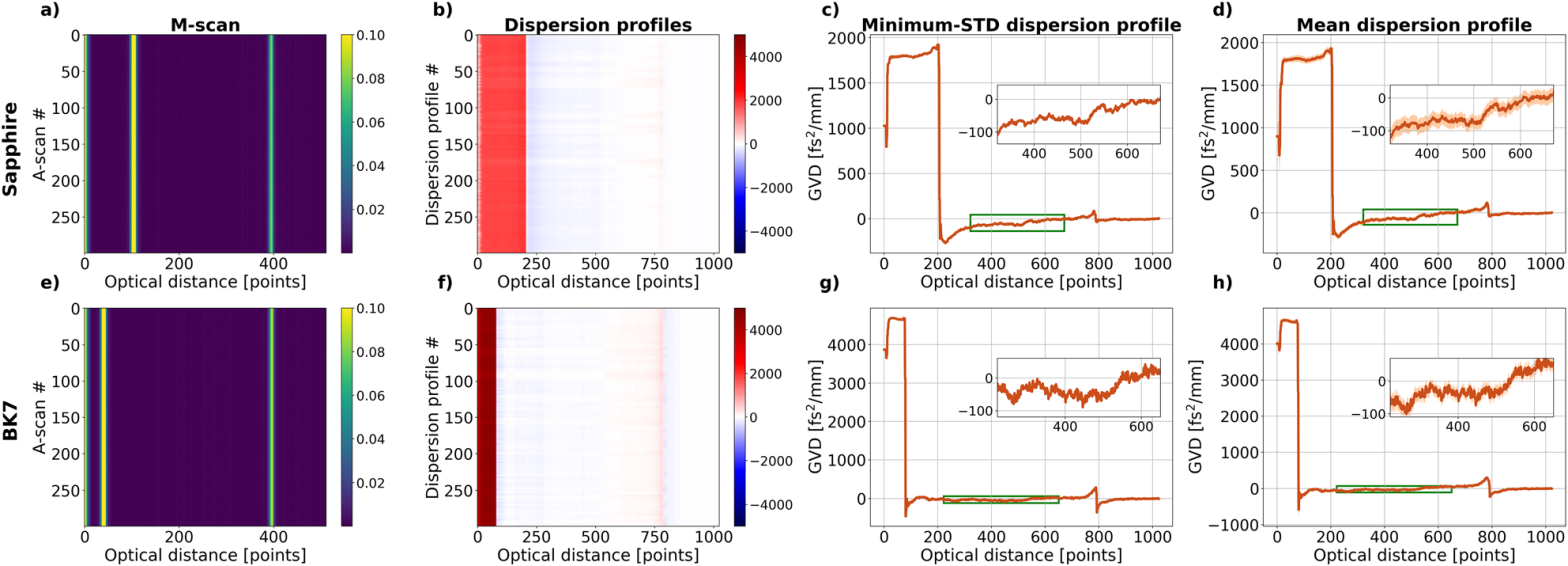
Sapphire and BK7: (**a**, **e**) an M-scan containing 300 A-scans with the autocorrelation peaks removed, (**b**, **f**) dispersion profiles corresponding to each A-scan in the M-scan, (**c**, **g**) dispersion profiles for which the standard deviation (STD) calculated from the GVD values in the green rectangle is the smallest, the inset is a closeup of the area in the plot marked with the green rectangle, (**d**, **h**) is the dispersion profile obtained by taking the mean of all the dispersion values, again the inset is a closeup of the area marked with the green rectangle. The twice bigger length of the x axis in the dispersion maps and dispersion profiles is a by-product of the FFT stack generation.

The neural network trained on signals with an SNR of 30 dB is used further to test if it is able to correctly determine the GVD values from the experimental data. Using the laboratory OCT system, we acquired 300 A-scans at one position for the 460-μm thick sapphire glass and a 1000-μm thick BK7 glass. Such A-scans, when stacked one on top of another, form an M-scan. The M-scan for the sapphire is shown in in Fig. 6a and the M-scan for the BK7 in Fig. 6e - in both cases the autocorrelation peaks were removed from every A-scan.

The corresponding GVD levels predictions are shown in Fig. 6b and f. The GVD value for sapphire at 840 nm is 53.2 fs$^2$/mm and the GVD value for BK7 is 40.98 fs$^2$/mm. Consequently, the GVD level in the area between the object interfaces for both the sapphire and BK7 should be very close to 0, resulting in light red colour in the dispersion map. That area in the obtained dispersion maps is predominantly light blue indicating that the GVD values are predominantly negative, which may be due to the reversed order of interfaces in the A-scan. When the order of interfaces is reversed in the A-scan, the first peak represents the back-surface of the object and the second peak represents the front surface. In such a situation, it is the first peak whose width is changed due to layer GVD which means that the accumulation of dispersion happens in the opposite, negative direction. Also, the GVD values in that area become positive at some point confirming what was established in the previous section: for smaller GVD values the network loses accuracy.

In Fig. 6c and g, we see dispersion profiles for which the standard deviation calculated from the GVD values in the range marked with the green rectangle is the smallest. Consequently, these dispersion profiles show the most “constant” GVD levels in all the acquired data. One can see in the bottom inset which shows the area inside the black rectangle, that still, the most “constant” GVD level varies in its height. The mean GVD value in such a case is calculated to be −46 ± 26 fs$^2$/mm for sapphire and −29 ± 27 fs$^2$/mm for BK7. As expected, both values are negative and burdened with a big error.

Figure 6d and h show a mean dispersion profile calculated from all the dispersion profiles on the corresponding maps. The light orange areas represent the standard deviation. The range of GVD values in the bottom inset, which shows the area inside the second green rectangle, is again broad. The mean of all the GVD values from all the dispersion profiles is calculated to be −50.3 ± 33.6 fs$^2$/mm for sapphire and −20.0±39 fs$^2$/mm for BK7. Again, as expected, both values are negative and burdened with a larger error than in the previous case.

In both cases, the mean GVD value is very close to the literature value for sapphire. For BK7, the mean values are further from the literature value, but remain within the calculated error.

### Dispersion maps of a grape and cucumber

**Figure 7:**
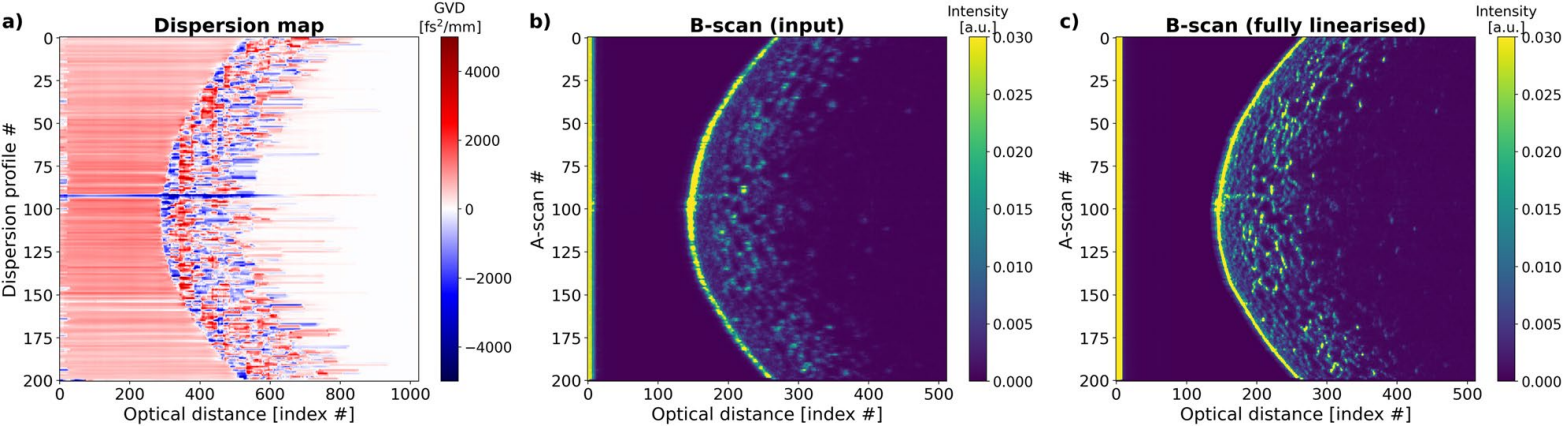
(**a**) Dispersion map of the grape, (**b**) the B-scan where the dispersion imbalance in the interferometer is not compensated. (**c**) An image for which the unbalanced dispersion was compensated. The twice bigger length of the x axis in the dispersion maps is a by-product of the FFT stack generation.

The dispersion maps are produced with the same neural network (trained with signals with SNR of 30dB) for biological objects: a grape (Fig. 7a), imaged with the laboratory OCT system, and a cucumber placed on a 120 μm thick cover glass, imaged with the commercial OCT system.

**Figure 8:**
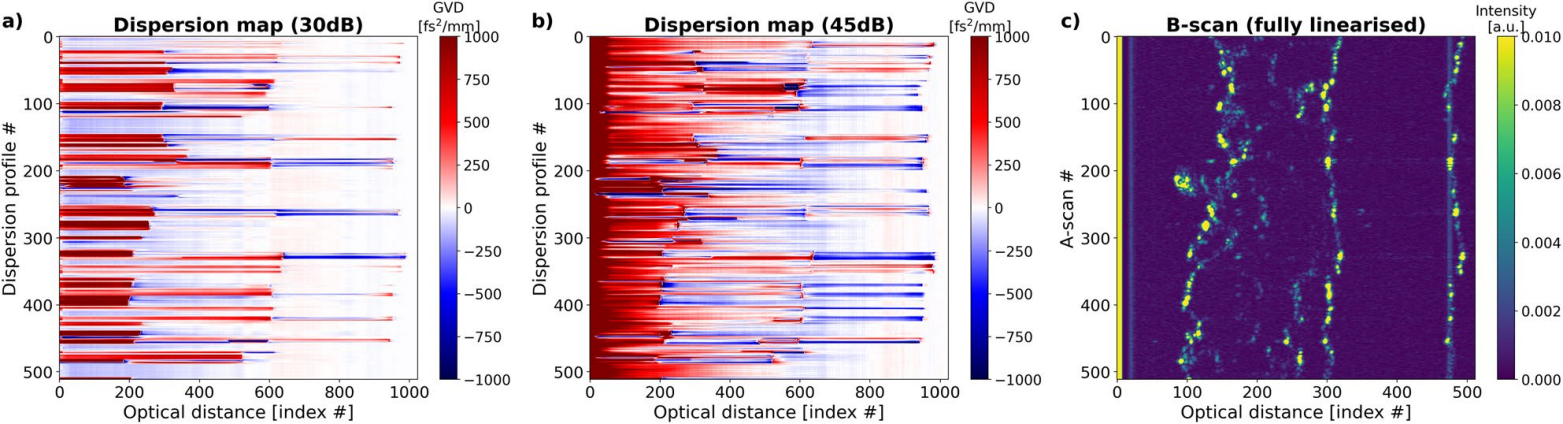
Dispersion maps of a cucumber obtained using the neural network trained with signals with (**a**) SNR = 30 dB, (**b**) and SNR=45dB show the boundaries of the imaged object, but the predicted GVD levels remain random. (**c**) The image of the cucumber. The twice bigger length of the x axis in the dispersion maps is a by-product of the FFT stack generation.

The predicted dispersion map of the grape shows a positive GVD area in front of the object, but the GVD levels for the inside of the grape remain random. Fig. 7b shows a B-scan obtained by Fourier transforming spectra used to calculate FFT stacks for the neural network: these spectra were linearised to only remove the nonlinearity coming from the spectrometer. In Fig. 7c, a B-scan is shown for which the spectra were fully linearised: the nonlinearities introduced by both the spectrometer and the unblalanced dispersion were removed.

The image of the cucumber (Fig. 8c) has a visibly lower SNR than the image of the grape. Since the intensity of both images are normalised, the comparison of the maximum values on the colourbars gives an indication on the difference in the level of noise with respect to the signal. For low-SNR input data, the dispersion maps, either obtained with the network trained with low-SNR signals (Fig. 8a) or high-SNR signals (Fig. 8b), are only able to distinguish the boundaries of the cucumber and the front of the cover glass on which the cucumber slice was placed on. For some A-scans, brighter elements inside the cucumber lead to the appearance of more dispersion levels in the dispersion profiles in those areas. However, the obtained values remain random.

## Summary and conclusions

Quantum-mimic OCT (Qm-OCT) and Machine Learning are combined to enable retrieval of Group Velocity Dispersion (GVD) values of imaged objects. With a Qm-OCT signal as input, the proposed VGG-16-based neural network allowed to obtain dispersion profiles for sapphire and BK7 glasses and, based on those dispersion profiles, calculation of GVD values. The calculated values, 46±26 fs$^2$/mm for sapphire and 29±27 fs$^2$/mm (original negative signs in front of the mean values are omitted) are in a good agreement with the literature values, 53.2 fs$^2$/mm for sapphire and 40.98 fs$^2$/mm for BK7, but are burdened with big errors. This is in contrast with current GVD-retrieving methods whose errors for well-defined glass structures could be as good as 0.3%^14^.

In the case of biological objects: a grape and a cucumber, the predictions are of rather qualitative nature. The predicted GVD values of their layers are very high or very low and change rapidly. This may be due to the small size of the layers and their small GVD values which in practice should be very close to the one of water, 26 fs$^2$/mm. A similar random GVD prediction was reproduced for computer-generated objects with thin, low-GVD layers and represents the biggest limitation of the presented approach.

Thin, low-GVD objects are out of reach of this approach, as evidenced by the simulations presented in Fig. 5, because thinner layers that are also characterised by a low GVD do not show changes in FFT stacks big enough for the network to pick up. What is important, this limitation is not restricted to quantum-mimic OCT and extends over to OCT in general. GVD manifests itself in a standard OCT spectrum by means of a nonuniform - nonlinear - modulation which after Fourier transformation corresponds to a broadened peak. In such a case, if the layer is small and has a low GVD, nonlinearity of the modulation (and the corresponding peak broadening) is also very small and consequently, challenging to detect, especially in the presence of noise. The accuracy of detecting such small changes is currently higher in other GVD-retrieving methods. However, the other methods require a close attention of the user, especially if one aims to characterise multiple layers. In comparison, our proposed approach provides a unique opportunity for GVD extraction to be fully automatic and user-unbiased. While the accuracy of this extraction is not fully satisfactory at the moment, there are no conceptual obstacles preventing it to be improved in the future.

Interestingly, the broad analysis presented in this article showed that the difficulty of GVD retrieval is not associated with the simplicity of the object or merely its thickness. As shown in Fig. 5, the success of accurate GVD determining increases for higher GVD values and their differences between adjacent layers in the object.

As mentioned before, the accuracy of determining GVD values with the proposed network is affected by noise in the signals. This is partially mitigated by a proper training, i.e. training performed using a dataset containing signals with an adequate noise level. In this respect, the future work should involve steps aimed at enhancing the training process. A second input could be added, for example, a traditional A-scan, which would support interface detection during training. Also different neural network architectures could be trialled and a more detailed simulation of an OCT signal could be employed, for example, one that includes the presence of speckles.

## Supplementary Information

Supplementary Information.

## Data Availability

The data used to support the findings of this study are included within the article. The datasets used during the current study are available from the corresponding author.
