# Supplementary material for: Extracting Group Velocity Dispersion values using quantum-mimic Optical Coherence Tomography and Machine Learning
Source: Sci Rep. 2023 Apr 22;13:6596. doi: 10.1038/s41598-023-32592-7 (PMC10122646; doi:10.1038/s41598-023-32592-7)
Supplement: Supplementary file 1 — Supplementary Information. [file 41598_2023_32592_MOESM1_ESM.pdf]

Extracting Group Velocity Dispersion values using quantum-mimic Optical Coherence Tomography and Machine Learning: supplemental document

This document discusses in detail the architecture, hyper-parameters and the choice of loss and output activation function of the model. Then, the overall performance is presented and analysed for training of models with datasets corresponding to different levels of noise. Finally, we provide an analysis showing which signal and object parameters play a key role in achieving a better goodness-of-fit of the predictions provided by our models.

1 Neural network

1.1 Preliminary model

Table SI Table 1 presents the list of hyper-parameters that were optimised with Optuna framework[1] in order to find the initial neural network for extracting Group Velocity Dispersion (GVD) values.

SI Table 1. List of optimised hyper-parameters and their optimal values. [A..B, C] - A and B are values of the limits of the range, and C is the step size.

Hyper-parameter	Values	Optimal
Type of pooling	max, avg	avg
Number of fully connected layers	[1..4, 1]	1
Units in each fully connected layer	[1024..16384, 1024]	14336
Dropout rates	[0.1..0.5, 0.05]	0.1
Learning rate	[1e-6, 1e-1]	2e-4
Optimizer	[Adam, Nadam, SGD, RMSProp]	Adam
Batch size	[16..128, 4]	16
Loss function	[Cosine Distance, Mean Squared Error, Mean Absolute Error (MAE)]	MAE

The optimised initial model architecture consists of batch normalization[2] layer after each convolutional layer and each convolutional block is followed by an average pooling layers , one fully connected layer with 14336 units, and a dropout rate[3] of 0.1 followed by a layer normalisation[4]. We trained this model with batch size 16, learning rate 0.0001, optimiser Adam[5], loss function Mean Absolute Error (MAE) and sigmoid as an output activation function.

1.2 Residual blocks

It has been shown[6] that incorporating residual blocks in VGG-16 architecture improves its performance. We tested several configurations of residual block integration within VGG-16. We found that instead of applying residual convolutional layers, a simple addition of the pooling layer and the convolutional output gives the lowest MAE loss.

The final model architecture is presented in SI Fig. 1. The purpose of an alpha layer (symbolised by a green dot with α character) is to expand the shape of average pooling output to match the shape of convolutional outputs before addition.

1.3 Output activation function

The network is trained on signals representing objects with GVD within the range [-5000, 5000] fs^2/mm. We normalise the GVD values to be within the range of [0,1], where 0 corresponds to -5,000 fs^2/mm, 1 - to 5,000 fs^2/mm, and 0.5 - to 0 fs^2/mm.

The retrieval of dispersion profiles from FFT stacks represents a regression problem that normally necessitates a linear activation in the output layer and then a normalisation layer to keep the model outputs within the range [0,1]. Instead, we decided to use a sigmoid activation function in the output layer because, surprisingly, it fits very well into our problem.

The sigmoid function (SI Fig. 2) returns 0.5 for the input argument value of 0, which reflects our situation in which the output 0.5 corresponds to the GVD equal to 0 fs^2/mm. Also, sigmoid converges to 1 for high positive input argument values and toward 0 for high negative ones. This trend is also seen in our case, 1 corresponds to high positive GVD values and 0 corresponds to high negative GVD values.

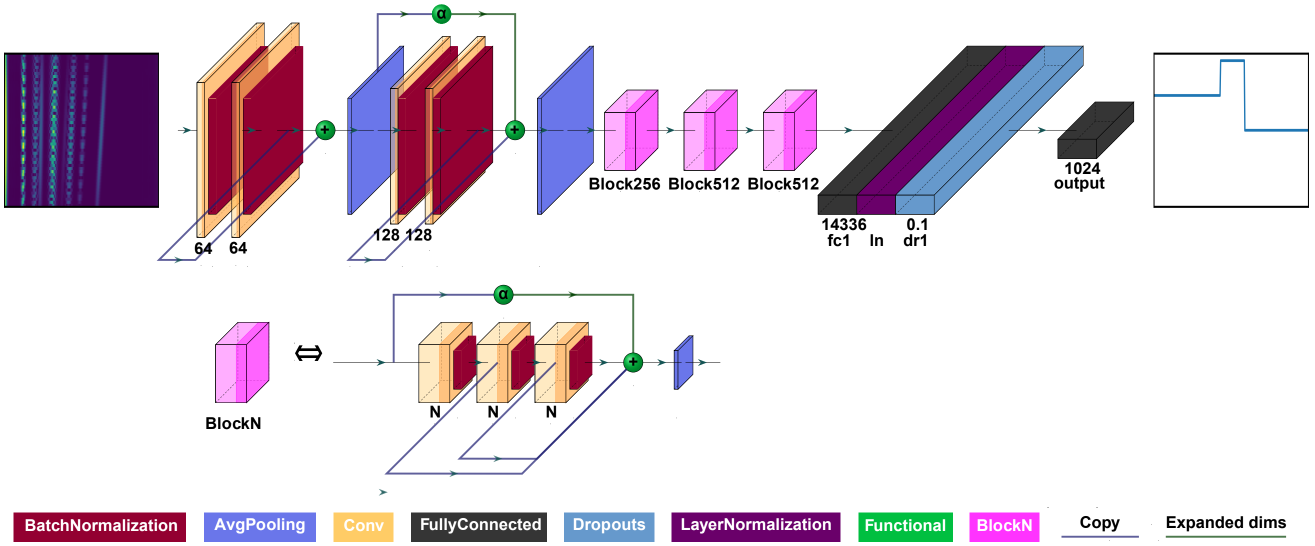

SI Figure 1. Model architecture. The blue lines are copies of the output of the layers. Green lines represent alpha layer (symbolised by the green dot with symbol) output with an expanded shape to match the shape of the convolutional layers inside the block. BlockN (pink square) is a block with convolutional layers each consisting of N filters. The plot was created with the help of PlotNeuralNet software?.

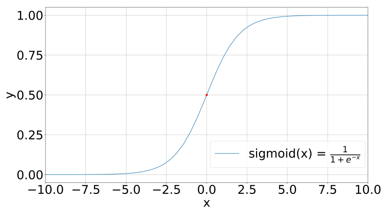

SI Figure 2. Sigmoid function plot

2 Metrics

We use Mean Absolute Error (MAE) as a loss function to measure the mean absolute differences between the true and predicted values during training. To determine how well our predictions fit the ground truths, we created three goodness-of-fit metrics that are presented in Algorithms 1-3.

The goodness-of-fit Algorithm 1 metric calculates the percentage of all prediction points being within the specified distance, dist, from the ground truth regardless of the GVD value. Algorithm 2 takes into account only ground truth values that are within the proximity of 0 fs^2/mm GVD, more specifically within the distance of 0.01 corresponding to 50 fs^2/mm, whereas Algorithm 3 calculates the goodness-of-fit for the GVD values outside of the -50 to 50 fs^2/mm range. We created the latter two metrics to be able to separately asses the performance of the models in the presence of small and big GVD values (see Subsection 4.2 Table SI Table 2).

During training and in our further analysis, we used the default values of GoFA, GoFAZL, and GoFOZL parameters.

Algorithm 1 Goodness-of-Fit Approximity (GoFA)

Input: ground truth vector $\mathbf{Y}$ with length N, prediction vector $\hat{\mathbf{Y}}$ with length N, maximum distance from $\mathbf{Y}$ $dist = 0.01$

d = |**Y** - $\hat{\mathbf{Y}}$|

for $i \leftarrow$ 1 to N **do**

$$\textbf{withinDist}_\text{i} = \begin{cases} 1 & \text{if } d_i < dist \\ 0 & \text{otherwise} \end{cases}$$

end for

GoFA = $\frac{1}{N}\sum$**withinDist**

Algorithm 2 Goodness-of-Fit Around Zero Level (GoFAZL)

Input: ground truth vector $\mathbf{Y}$ with length N, prediction vector $\hat{\mathbf{Y}}$ with length N, maximum distance from $\mathbf{Y}$ $dist = 0.01$, level of zero GVD value $zeroLvl = 0.5$

d = $|\mathbf{Y} - \hat{\mathbf{Y}}|$

for $i \leftarrow 1$ to N **do**

$$\mathbf{withinDist_i} = \begin{cases} 1 & \text{if } y_i < zeroLvl + dist \text{ and } y_i > zeroLvl - dist \\ 0 & \text{otherwise} \end{cases}$$

$$\mathbf{aroundZeroLvl_i} = \begin{cases} 1 & \text{if } d_i < dist \text{ and } withinDist_i = 1 \\ 0 & \text{otherwise} \end{cases}$$

end for

GoFAZL = $\frac{1}{N} \sum$ **aroundZeroLvl**

Algorithm 3 Goodness-of-Fit Outside Zero Level (GoFOZL)

Input: ground truth vector $\mathbf{Y}$ with length N, prediction vector $\hat{\mathbf{Y}}$ with length N, maximum distance from $\mathbf{Y}$ $dist = 0.01$, level of zero GVD value $zeroLvl = 0.5$

d = $|\mathbf{Y} - \hat{\mathbf{Y}}|$

for $i \leftarrow 1$ to N **do**

$$\mathbf{outsideDist_i} = \begin{cases} 1 & \text{if } y_i >= zeroLvl + dist \text{ or } y_i <= zeroLvl - dist \\ 0 & \text{otherwise} \end{cases}$$

$$\mathbf{outsideZeroLvl_i} = \begin{cases} 1 & \text{if } d_i < dist \text{ and } outsideDist_i = 1 \\ 0 & \text{otherwise} \end{cases}$$

end for

GoFOZL = $\frac{1}{N} \sum$ **outsideZeroLvl**

3 SNR analysis

We trained our model with several different datasets which represent a different level of noise. SI Fig. 3 presents the performance of the model after 100 epochs of training with datasets comprising signals with SNR of 25dB (SI Fig. 3 a,e), 30dB (SI Fig. 3 b,f), 35dB (SI Fig. 3 c,g), and 45dB (SI Fig. 3 d,h).

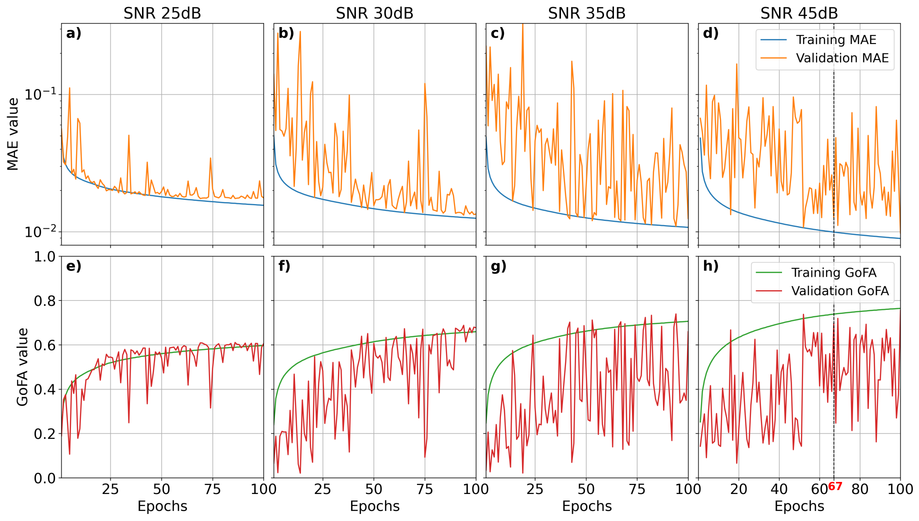

SI Figure 3. MAE and GoFA values after 100-epochs training with datasets incorporating different SNR levels, a,e) 25dB, b,f) 30dB, c,g) 35dB, d,h) 45dB. The dashed line in d,h shows the epoch from which we observe overfitting in the 45dB model.

Although in each case an overfitting seems to be observed, this is predominantly the problem for the 45dB dataset starting from epoch 67. From that epoch, the model becomes very unstable. For the 35dB dataset, the model also shows some minor instabilities, but it keeps improving over the course of the training. The 25dB model has no problems with instabilities. In case of the 30dB model, we observe a decrease in fluctuations and an overall improvement of the performance over time with no

overfitting at any epoch. Consequently, the higher the SNR of the training datasets, the less stable the training is. On the other hand, the higher the SNR, the better the predictions fit the true values as seen from the GoFA values in SI Fig. 3 e,f,g,h.

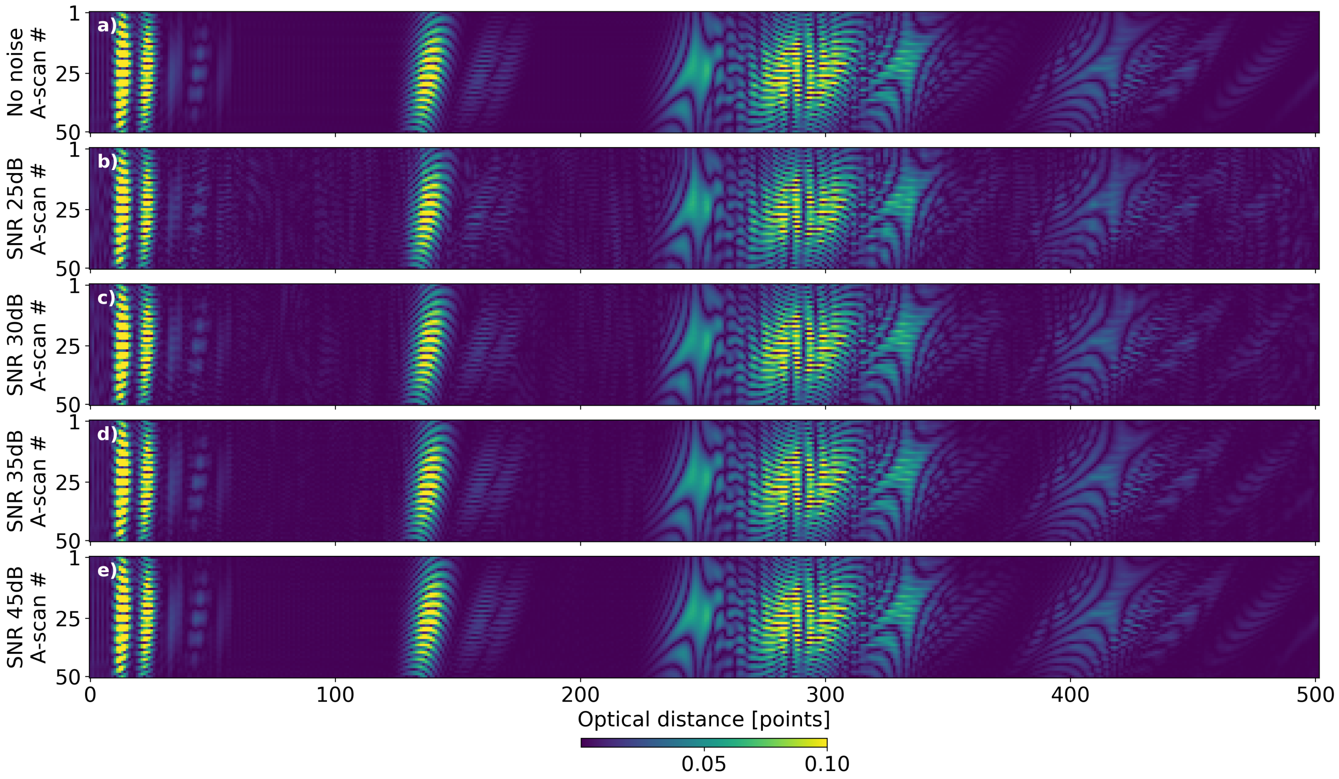

SI Figure 4. A random test input FFT stack with a different level of incorporated noise: a) without noise, b) SNR = 25dB, c) SNR = 30dB, d) SNR = 35dB, and e) SNR = 45dB. The inputs were trimmed to the first 512 points.

This behaviour is linked to how the noise changes the OCT signals and consequently the inputs of the networks. SI Fig. 4 shows a random test FFT stack with a different level of noise, SI Fig. 4b with 25dB, SI Fig. 4c with 30dB, SI Fig. 4d with 35dB, and SI Fig. 4e with 45dB. We present the same FFT stack for the noise-free signal in SI Fig. 4a. In SI Fig. 4b, we see that a high level of noise visibly deteriorates the FFT stack. We observe the appearance of additional elements and the degradation or removal of other information. The noise level corresponding to the SNR of 30dB has a detrimental effect on the FFT stack (SI Fig. 4c) but the changes in the structural information are barely visible. None of these effects is visible in SI Fig. 4e, which closely resembles a noise-free FFT stack (SI Fig. 4a).

To illustrate how SNR changes the predictions, we randomly selected several samples from the test dataset, each sample corresponding to a different number of interfaces, and calculated the predictions with the model for each SNR level. In each case, the training lasted for 100 epochs. SI Fig. 5 shows the comparison between predictions (orange lines) and ground truth (blue lines). SI Fig. 5 column 1) presents the data for training with dataset with SNR equal to 25dB, column 2) 30dB, column 3) 35dB, and column 4) 45dB. SI Fig. 5a) is a 2-interface object, b) a 4-interface object, c) a 8-interface object, and d) a 12-interface object.

SI Fig. 5 confirms previous statement that a higher SNR allows better predictions. Apart from the 45dB model being generally unstable, it provided very good results at the 100th epoch (SI Fig. 5 column 4), being the closest to the ground truths. Finally, we see that the predictions for the 25dB model (SI Fig. 5 column 1) are visibly further from the truth compared to other models.

It needs to be noted here that although higher SNR produces higher goodness-of-fit values, the model needs to perform well for a specific level of noise, i.e. the level of noise exhibited in the experimental data.

4 30dB model analysis

4.1 Metrics

Our further analysis was performed only on the model that provided the best results for our experimental data - the one trained with signals representing the SNR of 30dB. The analysis is based on a test dataset comprising 20,000 object samples that we

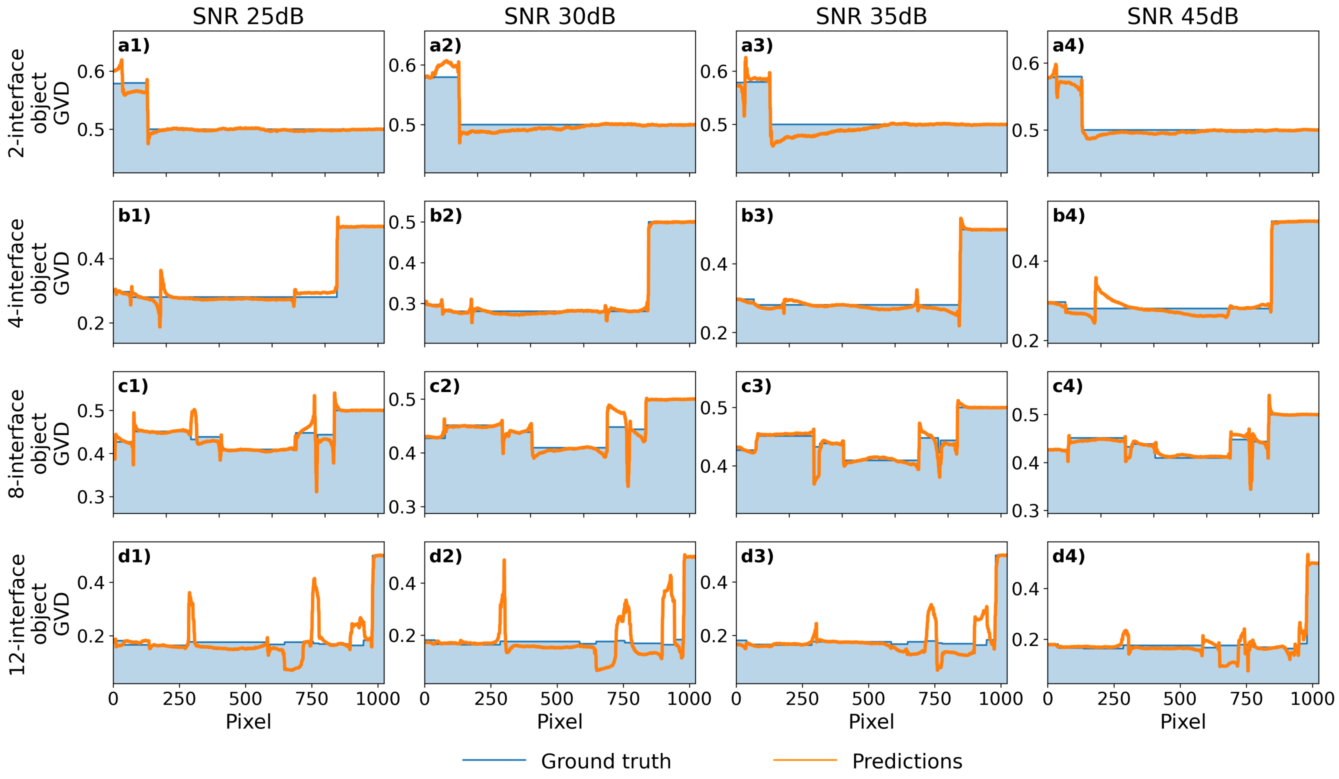

SI Figure 5. Comparison of ground-truth dispersion profiles (blue lines) and GVD predictions (orange lines) of random test samples. The objects had (row a) 2 interfaces, (row b) 4 interfaces, (row c) 8 interfaces, and (row d) 12 interfaces. Inputs incorporated different level of noise, (column 1) SNR = 25dB, (column 2) 30dB, (column 3) 35dB, (column 4) 45dB. Predictions were calculated with a model trained with corresponding noise level datasets. Each model was trained for 100 epochs.

created prior to training, ensuring that objects with the same structural information do not exist within training and test datasets. We further trained the model for another 100 epochs, resulting in 200 epochs in total.

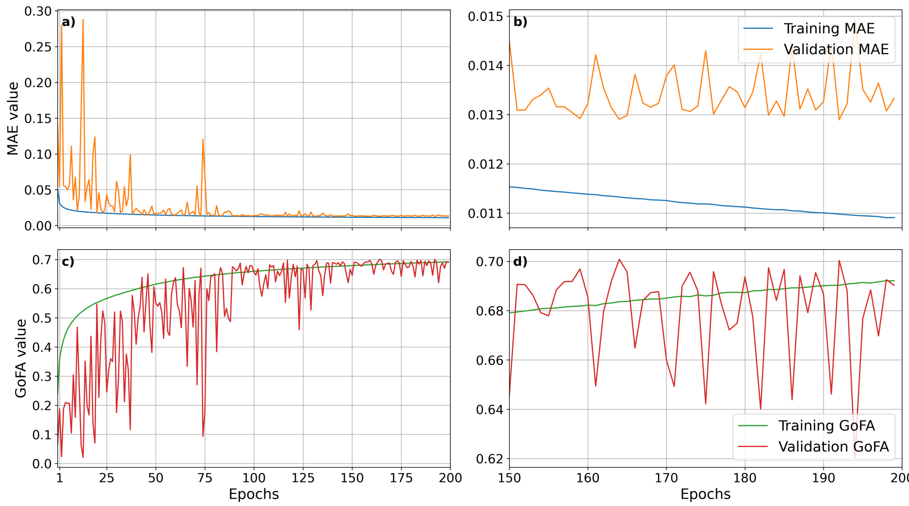

SI Figure 6. a,b) MAE and c,d) GoFAvalues after 200-epoch training SNR 30dB model. a,c) show the whole training process, b,d) show the training between 150th and 200th epoch.

SI Fig. 6 shows the performance of the model after 200-epoch-long training. We see that initial major fluctuations of loss and goodness-of-fit stop after 75 epochs and the model stabilises (SI Fig. 6a,c). In SI Fig. 6b,d, we observe a continuous

slight improvement of goodness-of-fit on the validation dataset until epoch 150 when validation MAE starts to slowly rise and validation GoFA reaches lower values giving the first signs of overfitting after epoch 190.

4.2 Number of interfaces and GVD variability

Our analysis shows that, in addition to SNR, the number of interfaces is another parameter that affects loss and goodness-of-fit. Table SI Table 2 presents the performance of the model for a different number of interfaces.

SI Table 2. Performance of 30dB SNR model values outside of the zero GVD for a test dataset. MAE (10^-3), GoFA, GoFAZL and GoFOTZL values are shown separately for each number of interfaces. "All" column shows the mean value of the metrics for all interfaces. Values in the brackets are standard deviation of the results. The best results are marked in bold.

Interfaces<br>Metrics	2	3	4	5	6	7	8	9
MAE [10^-3]	4.27 (2.12)	5.03 (3.01)	6.03 (3.94)	7.24 (4.95)	8.85 (5.89)	10.21 (6.31)	11.7 (6.58)	14.03 (7.9)
GoFA [%]	90.42 (10.51)	88.64 (11.61)	85.15 (13.96)	81.66 (14.42)	76.94 (16.15)	73.46 (16.32)	69.44 (16.59)	65.31 (16.41)
GoFAZL [%]	96.23 (7.88)	95.71 (9.55)	94.93 (11.94)	93.8 (14.23)	93.67 (14.35)	92.77 (15.27)	93.04 (14.09)	92.33 (15.44)
GoFOZL [%]	86.9 (15.72)	85.96 (14.65)	82.02 (16.57)	78.83 (16.02)	73.6 (18.25)	70.47 (17.73)	66.12 (18.14)	61.8 (17.83)

Interfaces<br>Metrics	10	11	12	All
MAE [10^-3]	16.11 (9.21)	18.41 (9.3)	20.59 (10.04)	11.27 (8.7)
GoFA [%]	61.21 (17.01)	56.56 (16.94)	53.29 (16.74)	72.65 (19.74)
GoFAZL [%]	91.18 (16.79)	90.53 (18.06)	89.7 (19.2)	93.05 (14.78)
GoFOZL [%]	57.64 (18.33)	52.94 (18.05)	49.92 (17.66)	69.39 (21.37)

We see a stark distinction between the results for objects with a high and a low number of interfaces. We notice that objects with numerous interfaces achieve a few times worse (=higher) MAE and significantly worse (=lower) GoFA, by up to 37%p between the highest GoFA for 2-interface objects and 12-interface objects. Although, according to the GoFAZL metric from Table SI Table 2, the model approximates quite well GVD close to 0 fs^2/mm for every number of interfaces and with over 89% goodness-of-fit, we see that the goodness-of-fit for the rest of the values (Table SI Table 2, GoFOZL metric) is much lower, from 49.9% for 12-interface objects to just 86.9% in the case of the simplest objects with 2 interfaces, and thus, values that are further from 0 fs^2/mm GVD have the greatest effect on the overall goodness-of-fit performance.

We visualised GoFA values for objects with a different number of interfaces in SI Fig. 7.

Among all test data samples, we did not observe one data sample with two interfaces that achieved 10-20% GoFA (SI Fig. 7d1). From the fact that we still see simplest objects with just two interfaces (SI Fig. 7a) with low GoFA, we deduced that there must be another factor besides SNR and the number of interfaces that affects the results.

We observed that the biggest deviations of predictions from the ground truth happens at the locations of the interfaces and the smaller the difference of GVD between the layers is, the worse the prediction of GVD gets. We checked how the spread of GVD within objects affects GoFA and presented the results in SI Fig. 8 for epoch 200.

The SI Fig. 8b-l confirm our previous conclusion that the number of interfaces has a major effect on GoFA. Predictions of simpler structures containing 2-4 interfaces (SI Fig. 8b-d) achieve higher mean GoFA results compared to the most complex object with 10-12 (SI Fig. 8j-l) which have the lowest GoFA values. On the same charts, we also see that standard deviation value of the GVD values within the object is another major factor that influences results. If we look at SI Fig. 8b, most examples with a high standard deviation, over 375 fs^2/mm, have the highest goodness-of-fit. Similar observation can be made regardless of the numbers of interfaces in the object - we obtained better GoFA results for objects whose interfaces' GVD values differ more. This observation is also confirmed in the analysis presented in Fig. 5 in the main article.

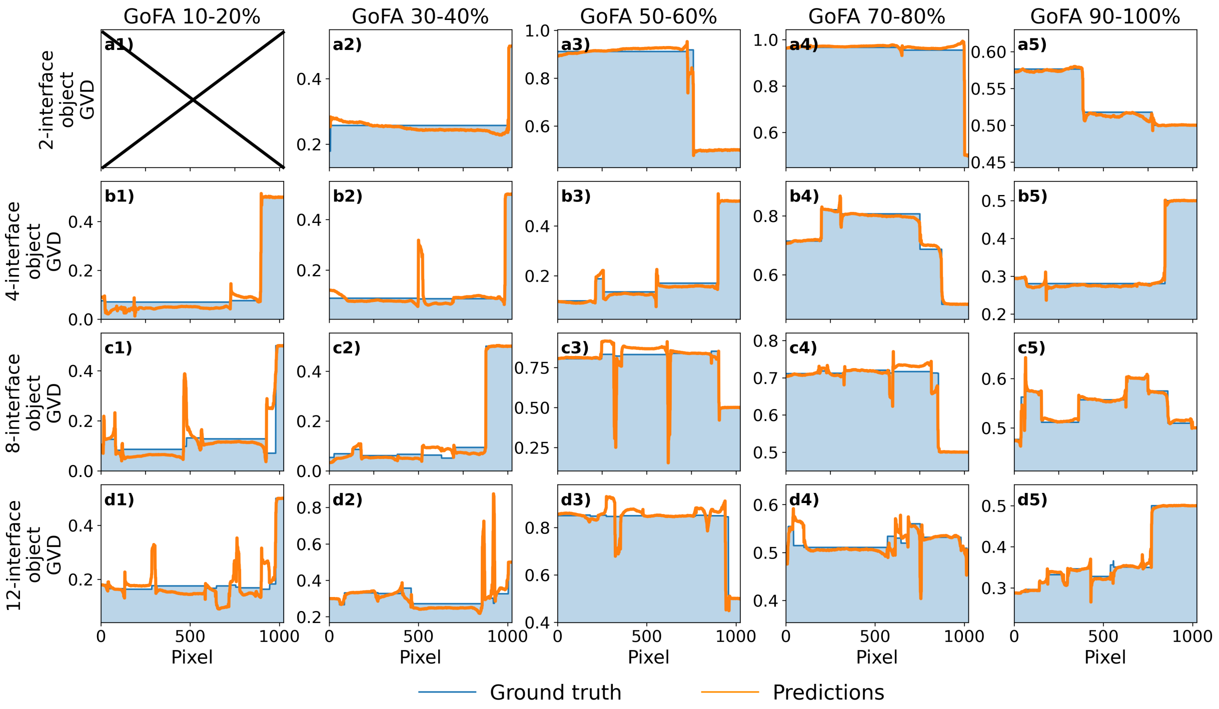

SI Figure 7. Comparison of ground-truth dispersion profiles (blue lines) and their predictions (orange lines) for the model trained with data corresponding to SNR equal to 30dB for objects with (row a) 2 interfaces, (row b) 4 interfaces, (row c) 8 interfaces, (row d) 12 interfaces, and for which the GoFA is in the range of (column 1) 10 to 20%, (column 2) 30-40%, (column 3) 50-60%, (column 4) 70-80%, (column 5) 90-100%. a1) There were no 2-interface objects that had GoFA in the range 10-20%.

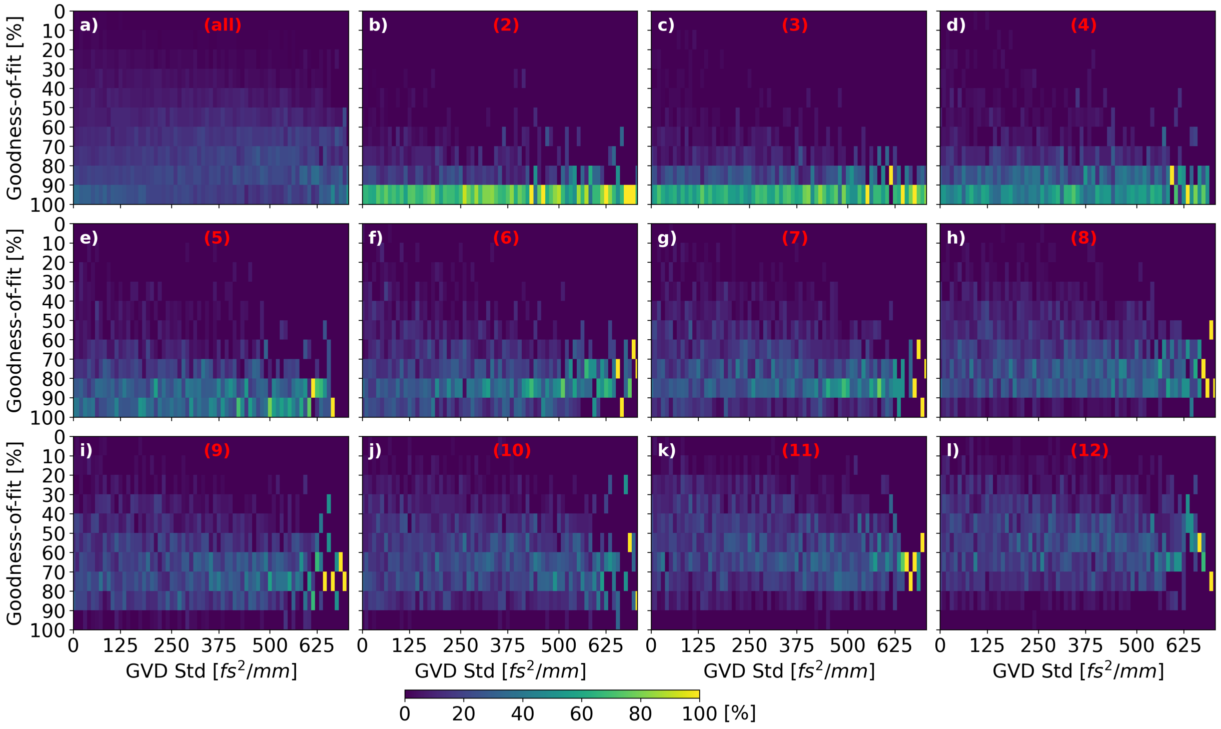

SI Figure 8. Percentage of test data samples with particular GoFA as a function of the number of interfaces in objects (the number is specified in red, all means the average result) and a standard deviation of interfaces' GVD that sum up to 100% for the particular column of the plot. Data for epoch 200.

References

1. Akiba, T., Sano, S., Yanase, T., Ohta, T. & Koyama, M. Optuna: A next-generation hyperparameter optimization framework. In *Proceedings of the 25th ACM SIGKDD international conference on knowledge discovery & data mining*, 2623–2631 (2019).

2. Ioffe, S. & Szegedy, C. Batch normalization: Accelerating deep network training by reducing internal covariate shift. In *International conference on machine learning*, 448–456 (PMLR, 2015).

3. Srivastava, N., Hinton, G., Krizhevsky, A., Sutskever, I. & Salakhutdinov, R. Dropout: a simple way to prevent neural networks from overfitting. *The journal machine learning research* **15**, 1929–1958 (2014).

4. Ba, J. L., Kiros, J. R. & Hinton, G. E. Layer normalization. *arXiv preprint arXiv:1607.06450* (2016).

5. Kingma, D. P. & Ba, J. Adam: A method for stochastic optimization. *arXiv preprint arXiv:1412.6980* (2014).

6. Chakraborty, I., Roy, D., Ankit, A. & Roy, K. Efficient hybrid network architectures for extremely quantized neural networks enabling intelligence at the edge. *arXiv preprint arXiv:1902.00460* (2019).
